# Effects of stand age on carbon storage in dragon spruce forest ecosystems in the upper reaches of the Bailongjiang River basin, China

**DOI:** 10.1038/s41598-019-39626-z

**Published:** 2019-02-28

**Authors:** Jianjun Cao, Yifan Gong, Jan F. Adamowski, Ravinesh C. Deo, Guofeng Zhu, Xiaogang Dong, Xiaofang Zhang, Haibo Liu, Cunlin Xin

**Affiliations:** 1 College of Geography and Environmental Science, Northwest Normal University, Lanzhou, 730070 China; 2 Department of Bioresource Engineering, Faculty of Agricultural and Environmental Science, McGill University, Sainte Anne de Bellevue, Québec, H9X 3V9 QC Canada; 3 School of Agricultural, Computational and Environmental Sciences, Centre for Sustainable Agricultural Systems & Applied Climate Sciences, University of Southern Queensland, Springfield, Central Qld 4300 Australia

## Abstract

At an ecosystem level, stand age has a significant influence on carbon storage (CS). Dragon spruce (*Picea asperata* Mast.) situated along the upper reaches of the Bailongjiang River in northwest China were categorized into three age classes (29–32 years, Y_1_; 34–39 years, Y_2_; 40–46 years, Y_3_), and age-related differences in total carbon storage (TCS) of the forest ecosystem were investigated for the first time. Results showed that TCS for the Y_1_, Y_2_, and the Y_3_ age groups were 323.64, 240.66 and 174.60 Mg ha^−1^, respectively. The average TCS of the three age groups was 255.65 Mg C ha^−1^, with above-ground biomass, below-ground biomass, litter, and soil in the top 0.6 m contributing 15.0%, 3.7%, 12.1%, and 69.2%, respectively. CS in soil and TCS of the Y_1_ age group both significantly exceeded those of the Y_3_ age group (*P* < 0.05). Contrary to other recent findings, the present study supports the hypothesis that TCS is likely to decrease as stand age increases. This indicates that natural resource managers should rejuvenate forests by routinely thinning older stands, thereby not only achieving vegetation restoration, but also allowing these stands to create a long-term carbon sink for this important eco-region.

## Introduction

Covering roughly 4.0 × 10^8^ km^2^ (30.8%) of the earth’s land surface in 2015^1^, forested land dominates the earth’s terrestrial ecosystems. Besides their key role in supplying timber^2^, forests have generated considerable attention for their primal role in the functioning, productivity and sustainability^3^ of the global ecosystem as well as in the protection of soil and restoration of landscapes^4^. Forests also play a special role in mitigating atmospheric CO_2_ concentrations. This is especially significant considering that about two-thirds of the terrestrial ecosystems’ organic carbon stocks are in forests, of which 81% is soil storage and 19% is plant storage^5,6^. However, forest landscapes have been significantly modified by human activities over hundreds of years^7^, leading to concern about restoration.

With the global implementation of forest landscape restoration to balance different functions at the landscape scale including water regulation, wildlife habitats, biodiversity and carbon storage (CS)^8^, the CS capacity of forest stands based on type and age has been studied extensively^9,10^. However, studies more often focus on carbon storage in soil (SCS) than on CS at the ecosystem level. At the ecosystem level, there are generally three interconnected carbon pools, namely live biomass, dead biomass, which plays an important role between soil carbon and biomass carbon, and organic soil horizons^11,12^. Based on a few ecosystem level studies on CS^2,9,13–18^, we found that forests in the latter stages of stand development could either be carbon neutral^19–21^, sequester a small quantity of carbon^22^, or exhibit a declining carbon pool^23^. This indicates that the dependent relationship between total forest ecosystem carbon storage (TCS) and stand age may be species- and site-specific^24,25^.

Dragon spruce (*Picea asperata* Mast.) is one of the preferentially planted trees for water and soil conservation in the upper reaches of the Bailongjiang River, China, and the present research aims to investigate the influence of stand age, focusing particularly on CS at the ecosystem level. With the knowledge gained from this investigation, the connection between plantation forestry strategy and CS is likely to be better understood^19^, allowing for more informed decisions^26^ on the management of current plantation practices in the region. The hypotheses of this study are: (1) stand age may have different influences on CS within live biomass, dead biomass, and organic soil horizons due to positive feedback between plants and soil, and (2) TCS is inversely proportional to stand age.

## Results

### Plant and soil properties under different age groups

With regard to tree growth, it was observed that diameter at breast height (DBH) was significantly greater in the Y_3_ age group compared with the other two groups (P < 0.05), but stand density (SD) was significantly greater in the Y_1_ age group compared to that in the Y_3_ age group (P < 0.05; Table 1). There was no significant difference in canopy density (CD) among the three groups (P > 0.05). Although the above- and below-ground biomass of trees, shrubs, and litter biomass were smaller in the Y_1_ and Y_2_ age groups than in the Y_3_ age group, the above- and below-ground biomass of herbs was greater in the Y_3_ age group, and there were no statistically significant differences between the three age groups for above- and below-ground biomass and dead biomass (P > 0.05, Table 1).

**Table 1:** Differences of plants and soil physical properties by stand age (mean ± standard deviation).

Measured variables			Stand designation and age		
			Y_1_ 29–32 years	Y_2_ 34–39 years	Y_3_ 40–46 years
Plants		DBH (cm)	15.42 ± 1.96^a^	16.43 ± 2.09^a^	19.82 ± 2.96^b^
		SD (tree ha^−1^)	1425.67 ± 306.41^a^	1134.34 ± 394.12^ab^	804.67 ± 63.96^b^
		CD (%)	0.77 ± 0.08^a^	0.76 ± 0.10^a^	0.70 ± 0.00^a^
	Above-ground biomass	Trees (10^4^ kg ha^−1^)	7.36 ± 1.19^a^	7.37 ± 2.63^a^	7.29 ± 3.72^a^
		Shrubs (10^4^ kg ha^−1^)	0.61 ± 0.92^a^	0.69 ± 0.66^a^	0.25 ± 0.18^a^
		Herbs (10^4^ kg ha^−1^)	0.16 ± 0.03^a^	0.20 ± 0.08^a^	0.23 ± 0.04^a^
		TBM_ag_ (10^4^ kg ha^−1^)	8.13 ± 1.59^a^	8.26 ± 2.65^a^	7.77 ± 3.52^a^
	Dead biomass	Litter (10^4^ kg ha^−1^)	7.20 ± 1.93^a^	6.18 ± 1.82^a^	6.95 ± 0.97^a^
	Below-ground biomass	Trees (10^4^ kg ha^−1^)	1.47 ± 0.24^a^	1.47 ± 0.53^a^	1.46 ± 0.74^a^
		Shrubs (10^4^ kg ha^−1^)	0.36 ± 0.54^a^	0.40 ± 0.39^a^	0.15 ± 0.11^a^
		Herbs (10^4^ kg ha^−1^)	0.13 ± 0.03^a^	0.16 ± 0.07^a^	0.19 ± 0.04^a^
		TBM_bg_ (10^4^ kg ha^−1^)	1.96 ± 0.62^a^	2.04 ± 0.63^a^	1.79 ± 0.63^a^
Soil		pH	7.39 ± 0.53^a^	7.57 ± 0.44^a^	7.84 ± 0.21^a^
		*θ* (%)	43.03 ± 16.16^a^	34.08 ± 16.36^b^	30.96 ± 11.85^b^
		*ρ* (Mg m^−3^)	1.11 ± 0.14^a^	1.24 ± 0.22^ab^	1.45 ± 0.05^b^
		*f* (%)	58.50 ± 5.32^a^	53.45 ± 8.07^ab^	45.33 ± 2.08^b^
		SOC (g kg^−1^)	37.46 ± 24.41^a^	23.00 ± 22.28^b^	11.10 ± 6.62^b^
		TN (g kg^−1^)	2.98 ± 0.96^a^	1.87 ± 1.46^ab^	0.83 ± 0.24^b^
		TP (g kg^−1^)	2.00 ± 0.98^a^	1.32 ± 0.81^ab^	0.75 ± 0.06^b^
		TK (g kg^−1^)	32.39 ± 4.87^a^	33.13 ± 5.89^a^	28.74 ± 2.14^a^
		AN (mg kg^−1^)	206.62 ± 51.95^a^	146.85 ± 109.39^ab^	69.38 ± 19.22^b^
		AP (mg kg^−1^)	1.94 ± 0.91^a^	1.81 ± 1.23^a^	1.23 ± 0.63^a^
		AK (mg kg^−1^)	218.31 ± 92.24^a^	200.61 ± 103.6^a^	139.99 ± 41.30^a^

Different letters indicate differences by stand age (row-wise) at *P* < 0.05. Where CD = canopy density; SD = stand density; DBH = diameter at breast height; *θ* = soil water content; *ρ* = soil bulk density; *f* = soil porosity; SOC = soil organic carbon; TN = total nitrogen; TP = total phosphorus; TK = total potassium; AN = alkaline hydrolysis nitrogen; AP = available phosphorus; AK = available potassium; TBM_ag_ and TBM_bg_ = total above- and below-ground biomass.

**Figure 1:**
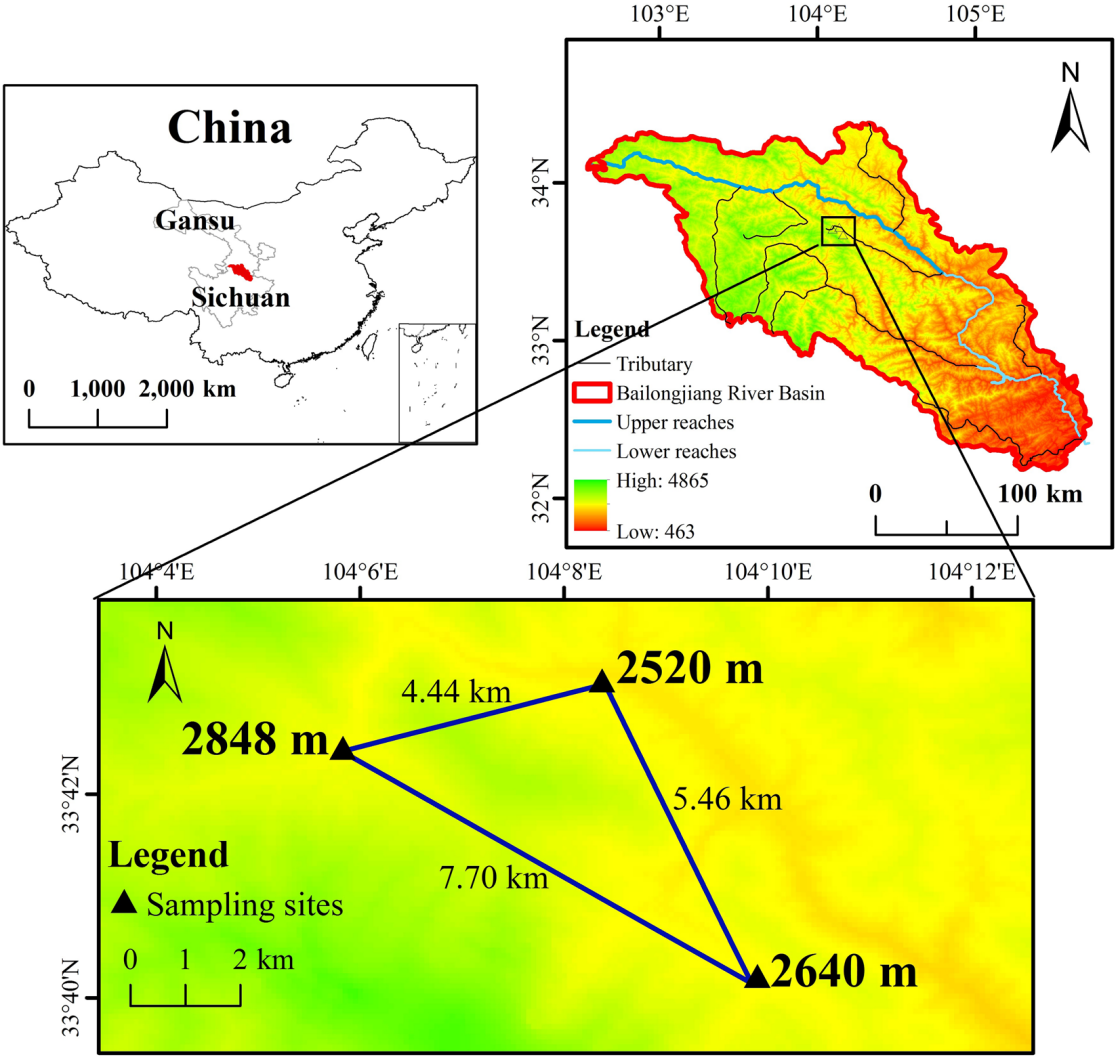
The distribution of soil organic carbon (SOC) by stand age and by soil layer. Different letters indicate differences at *P* < 0.05 level. Capital letters: difference in stand age. Lowercase letters: differences among soil depths.

No significant difference in pH was measured among the three age groups (*P* > 0.05). In the Y_1_ age group soil bulk density (*ρ*) was significantly lower while soil moisture content (*θ*) and soil porosity (*f*) were relatively higher (*P* < 0.05) than the other two age groups. Total nitrogen (TN), total phosphorus (TP), and alkaline hydrolysis nitrogen (AN) were significantly higher in the Y_1_ group (2.98 g kg^−1^, 2.00 g kg^−1^, and 206.62 mg kg^−1^, respectively) compared to those in the Y_3_ age group (0.83 g kg^−1^, 0.75 g kg^−1^, and 69.38 mg kg^−1^, respectively). Total potassium (TK), available phosphorus (AP), and available potassium (AK) had similar values among all three age groups. The average soil organic carbon (SOC) of the 0–0.6 m soil depth was substantially greater in the Y_1_ age group (37.46 g kg^−1^) compared to the Y_2_ (23.00 g kg^−1^) and the Y_3_ (11.10 g kg^−1^) age groups (*P* < 0.05, Table 1; Fig. 1).

### Vertical distribution of SOC in different age groups

**Figure 2:**
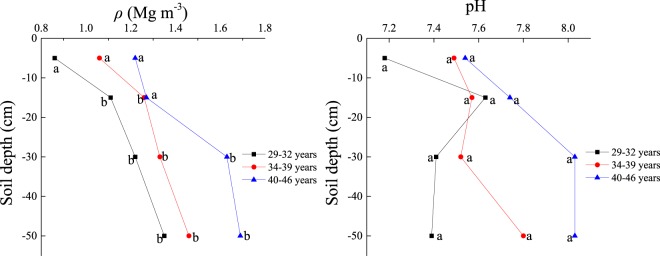
Biplot of the first two PCA axes of biological factors, soil factors, and the three stand age ranges. CD = canopy density; SD = stand density; TBM_ag_ and TBM_bg_ = total above- and below-ground biomass; DBH = diameter at breast height; *θ* = soil moisture content; *ρ* = soil bulk density; *f* = soil porosity; TN = total nitrogen; TP = total phosphorus; TK = total potassium; AN = alkaline hydrolysis nitrogen; AP = available phosphorus; AK = available potassium; TC_BMag_, TC_BMbg_ and C_litter_ = carbon storage in TBM_ag_, TBM_bg_ and litter; SCS = soil carbon storage; TCS = forest ecosystem total carbon storage.

Decreasing SOC with increasing soil depth was observed across all age groups (Fig. 2). The greatest SOC in the 0–0.1 m top soil was found in the Y_1_ age group (60.5 g kg^−1^), while the smallest value (20.8 g kg^−1^) was found in the Y_3_ age group. In both these age groups, there were no significant differences in SOC values among the three deeper soil layers (P > 0.05). In the Y_2_ age group, from the 0–0.1 m to the 0.1–0.2 m soil depth, SOC levels decreased from 38.99 g kg^−1^ to 23.34 g kg^−1^, but no significant difference was found between them (P > 0.05). However, SOC in the 0–0.1 m soil layer was significantly higher than that of the 0.2–0.4 m and 0.4–0.6 m soil layers (P < 0.05), with no significant difference between the latter two soil layers (P > 0.05).

### Carbon pools at different elevations and age groups

The highest value of TCS was 312 Mg ha^−1^ at the 2520 m elevation, significantly higher than the 204 Mg ha^−1^ observed at the 2640 m elevation (P < 0.05), but not different from the 249 Mg ha^−1^ observed at the 2848 m elevation (P > 0.05). Although there was variation in TCS between the 2640 m and 2848 m elevations, this difference was not statistically significant (P > 0.05).

TCS and SCS trended negatively with stand age, as there were significant differences between the Y_1_ and the Y_3_ age groups for both variables (*P* < 0.05; Table 2). Other carbon pools did not change significantly with increasing stand age (*P* > 0.05), although they presented a decreasing trend. SCS was the largest carbon pool in the forests, accounting for up to 69.2% of the TCS. CS in total above-ground biomass (TC_BMag_) and in total below-ground biomass (TC_BMbg_) contributed 15.0% and 3.7%, respectively, while carbon storage in litter (C_litter_) made up 12.1% of the TCS (Table 2).

**Table 2:** Magnitude of CS (Mg ha^−1^), mean ± standard deviation in different age groups, and average percentage contribution to total forested ecosystem carbon storage.

	Age range						Mean	%
	Y_1_ 29–32 years	%	Y_2_ 34–39 years	%	Y_3_ 40–46 years	%		
TC_BMag_	38.80 ± 7.57^a^	12.0	38.68 ± 12.26^a^	16.1	37.06 ± 16.83^a^	21.2	38.47 ± 11.15	15.0
TC_BMbg_	9.37 ± 2.97^a^	2.9	9.57 ± 2.91^a^	4.0	8.55 ± 2.99^a^	4.9	9.36 ± 2.80	3.7
C_litter_	33.83 ± 9.08^a^	10.5	29.05 ± 8.54^a^	16.2	32.65 ± 4.55^a^	18.7	31.02 ± 8.21	12.1
SCS	241.64 ± 68.81^a^	74.7	163.36 ± 98.13^ab^	67.9	96.34 ± 19.39^b^	55.2	176.79 ± 93.85	69.2
TCS	323.64 ± 77.03^a^	100	240.66 ± 100.38^ab^	100	174.60 ± 12.44^b^	100	255.65 ± 97.51	100

Different letters indicate differences at *P* < 0.05. TC_BMag_ and TC_BMbg_ = carbon storage in total above- and below-ground biomass; C_litter_ = carbon storage in litter; SCS = soil carbon storage; TCS = total forested ecosystem carbon storage.

### Relationships between the measured variables

Principal component analysis (PCA) revealed that the first two PCs (i.e., PC1 and PC2) accounted for 40.13% and 21.45%, respectively, of the total variance of data obtained in the 21 study plots (Table 3). This indicated that the first two principal components accounted for 61.58% of the standardized variance. As evident in Fig. 2, the PCA generated two distinct clusters, with soil characteristics forming one cluster and forest characteristics forming the other, which were well discriminated between the Y_1_ and the Y_3_ age groups. Physical and chemical soil properties contributed predominantly to PC1, whereas forest characteristics contributed predominantly to PC2.

**Table 3:** Eigenvalues and contributions based on principal component analysis (PCA).

Component	Eigenvalues	Contribution rate (%)	Cumulative contribution rate (%)
1	9.23	40.13	40.13
2	4.93	21.45	61.58
3	2.56	11.13	72.71
4	1.56	6.80	79.51
5	1.27	5.52	85.03
6	1.11	4.80	89.84

Significant negative correlations appeared between SD and DBH, and CD and DBH, whereas significant positive correlations were found between SD and CD, and litter and CD (*P* < 0.05). The *θ* had a significant negative correlation with *ρ*, but a significant positive correlation with *f*, SOC, TN, TP, AN and AP (*P* < 0.05). The *ρ* had a significant positive relationship with DBH and a significant negative relationship with SD, *f*, SOC, TN, TP, AN and AP (*P* < 0.05), while *f* exhibited the opposite trend. In addition, SOC had significant positive relationships with TN, TP, AN and AP (*P* < 0.05) (Table 4).

**Table 4:** Relationships between measured variables assessed on the basis of Pearson’s correlation r value.

	SD	DBH	CD	TBM	Litter	*θ*	*ρ*	*f*	pH	SOC	TN	TP	TK	AN	AP	AK
SD	1															
DBH	−0.63^**^	1														
CD	0.63^**^	−0.54^*^	1													
TBM	0.54^*^	0.02	0.39	1												
Litter	0.42	−0.38	0.59^**^	0.33	1											
*θ*	0.45^*^	−0.44	−0.09	0.01	−0.03	1										
*ρ*	−0.54^*^	0.46^*^	0.03	−0.03	0.04	−0.84^**^	1									
*f*	0.53^*^	−0.47^*^	−0.04	0.02	−0.05	0.84^**^	−1.00^**^	1								
pH	−0.24	0.41	−0.22	−0.08	−0.29	−0.19	0.08	−0.10	1							
SOC	0.43	−0.32	−0.22	0.10	0.01	0.89^**^	−0.83^**^	0.82^**^	−0.19	1						
TN	0.39	−0.26	−0.22	0.13	0.03	0.71^**^	−0.77^**^	0.77^**^	−0.25	0.94^**^	1					
TP	0.35	−0.10	−0.35	0.10	−0.26	0.55^*^	−0.72^**^	0.72^**^	−0.13	0.77^**^	0.86^**^	1				
TK	0.17	−0.09	0.03	0.15	0.20	0.13	−0.20	0.18	0.02	0.38	0.43	0.40	1			
AN	0.37	−0.25	−0.30	0.001	−0.14	0.83^**^	−0.88^**^	0.88^**^	−0.16	0.94^**^	0.90^**^	0.81^**^	0.19	1		
AP	0.16	−0.27	−0.15	−0.08	0.03	0.67^**^	−0.60^**^	0.60^**^	−0.19	0.63^**^	0.55^*^	0.27	−0.23	0.71^**^	1	
AK	0.09	−0.05	−0.16	−0.05	0.14	0.33	−0.29	0.29	0.18	0.40	0.27	0.11	0.15	0.37	0.19	1

**^,^*Significant at *P* < 0.01, *P* < 0.05, respectively. CD = canopy density; SD = stand density; TBM = total above-ground biomass + total below-ground biomass; DBH = diameter at breast height; *θ* = soil water content*; ρ* = soil bulk density; *f* = soil porosity; SOC = soil organic carbon; TN = total nitrogen; TP = total phosphorus; TK = total potassium; AN = alkaline hydrolysis nitrogen; AP = available phosphorus; AK = available potassium.

## Discussion

### Vertical distribution of SOC across forest age

**Figure 3:**
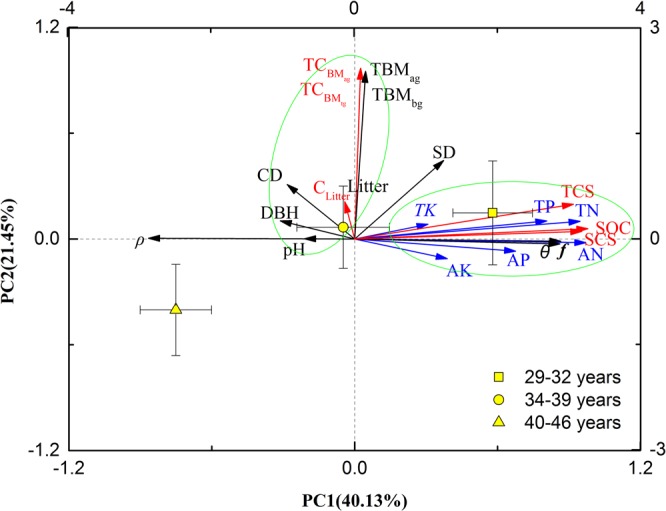
The vertical distribution of soil pH and ρ in each age group. Different letters indicate differences among soil depths at *P* < 0.05 level.

In accordance with other forest ecosystem studies^27,28^, this study showed that SOC generally decreased with increased soil depth. SOC in the 0–0.1 m soil layer was the highest in each age group because this layer incorporated ground litter and created organic matter through bioturbation^27^, whereas, at a depth of 0.4–0.6 m, there was a significantly lower level of SOC (Table 1) due to root distribution^29^. The vertical distributions of soil pH and *ρ* also influenced the vertical distribution of SOC. The present study showed a trend of increasing soil pH and *ρ* with the soil depth in each age group (Fig. 3), but SOC exhibited the opposite trend, as low soil pH and *ρ* are more favorable to an accumulation of SOC^27^. Generally, lower soil pH could enhance the availability of micronutrients, such as copper, iron, and manganese, which are important for root growth^30^. Also, the low *ρ* indicated a better soil structure, and thus can stabilize soil organic matter by soil particles and associated iron oxides^31,32^.

### Variation of plant, soil and TCS along with the forest age

No differences in TBM_ag_ and TBM_bg_ were observed among the three age groups, which concurred with results from studies by Frouz *et al*.^33^ and Ligot *et al*.^34^. This was likely due to SD decreasing as DBH increased with stand age (Table 1). Generally, larger trees had high individual growth rates, but biomass production decreased with the abundance of larger trees in comparison to that of small trees^34^. However, this result was contrary to the studies of He *et al*.^35^ and Dai *et al*.^13^. This disparity was likely due to the primary forest samples used in these previous studies, which often contained more native species, greater biodiversity, and higher biomass density than plantation forests. In the present study, stable litter levels indicated that litter was almost unaffected by stand age, as confirmed by Rodin and Bazilevich^36^. This is consistent with the concept that, as trees continue to age, resources are allocated only for maintenance and survival^19,35^, limiting biomass^34^ and therefore, litter production.

Soil pH is primarily determined by litter, which can create more acidic soil due to organic matter decomposition^27^, and by tree evaporation and transpiration, which can result in a higher soil pH^37^. In the present study, there was no difference in soil pH among the three age groups, which is inconsistent with previous studies^27^. This may suggest that the differences in litter, tree evaporation and transpiration among the three stand age groups were insufficient to cause pH variation. The *ρ* increased with stand age, but *f* presented an inverse trend. This might have resulted from a decrease in the soil’s biological activities due to declining root penetration^33^ and an increase in anthropogenic soil compaction^38^. The *θ* decreased as stands aged as confirmed by others^39–41^; older trees consume more soil water due to more intensive evapotranspiration. In some cases, older trees may have a larger understory composed of grasses and this can also cause more soil water consumption. This was not the case in the present study as there were no differences in understory biomass among the three age groups (Table 1).

It is important to assess the soil carbon budget with respect to plantation age^9,42^. Generally, a high SOC is likely to maintain and improve soil fertility and quality^43^. Based on the three age groups investigated in the study area, the average SOC decreased with stand age (Table 1, Fig. 2). This result coincided with the results of Dangal *et al*.^19^, and was due to an increase in the rate of organic matter decomposition and variation in the soil’s hydrothermal regime for maintaining tree survival. TN, AN and TP were found to be significantly lower in the Y_3_ age group than in the Y_1_ age group, but TK, AP and AK were similar (Table 1), which was consistent with Fan *et al*.^44^. This was explained by the amount of litter, which tended to decrease with stand age and would thus release fewer soil nutrients from the litter into the soil^45^. Combined with other indicators such as the tendency for plant root mass to decrease with stand age (Table 1), it is conceivable that soil nutrients from these sources also diminished with stand age^46,47^. In addition, as *f* and *θ* decreased with stand age, higher soil respiration occurred^43^ in the Y_3_ age group, resulting in more soil nutrients being consumed by soil microbes and roots. Finally, as AP was mainly affected by the decomposition of litter, and TK and AK were affected by the soil parent material and the stability of its properties^45^, no significant differences were found among the three age groups.

SCS in the Y_1_ age group was significantly higher than that in the Y_3_ age group (Table 2), suggesting that dragon spruce may continue to decline in productivity with age, as seen in other tree species^19,35^. The average SCS (176.79 Mg ha^−1^) in this study (Table 2) was lower than the forest average for SCS across China estimated by Zhou *et al*. (194 Mg C ha^−1^)^48^, but was higher than that estimated by Tang *et al*. (126 Mg C ha^−1^)^49^. The latter authors took the spatial discrepancies of soil depths and soil gravel content into account when assessing SCS. Differences in SCS among the three age groups in the current study were inconsistent with the results of Dai *et al*.^13^, which showed a significant positive relationship between SCS and stand age.

### Factors influencing SOC and TCS of the forest

According to the results of the PCA (Fig. 2), the influence of TBM_ag_, TBM_bg_, and litter biomass on SOC was negligible while the soil properties were the major influencing factors. In forest lands, most previous studies found that soil pH was negatively related to SOC (e.g.^30,50^). However, soil pH was not related to SOC within the study area (Table 4) as supported by Wang *et al*.^51^ in *Larix gmelinii* plantations in northeast China. This suggests that the relationship between soil pH and SOC requires further research. The relationships between SOC and *ρ*, *f* and *θ* (Table 4) could be explained by soil permeability; with greater soil permeability comes more favorable water infiltration and thus SOC can be increased by increasing decomposition and input of litter^52,53^, enhancing the growth rate of plants^47^. In general, higher *ρ* indicates poor soil permeability and results in reduced carbon mineralization^46^. However, Wang *et al*.^54^ found a significant positive correlation between SOC and *ρ*, suggesting that the mechanisms linking these two variables must be explored further. Moreover, soil respiration, an important indicator of soil quality and soil fertility, often decreases with an increase of *θ*^43,55^. In the present study, *θ* decreased as stands aged (Table 1), which might have led to higher soil respiration, in turn causing a decrease in SOC. A previous study in central Ireland, however, found that soil respiration of dragon spruce stands showed a decreasing SOC trend with increasing stand age^56^. This suggests that the relationship between soil respiration and stand age may depend on the specific regional climate and on human activities.

Except for TK and AK, the relationship between other soil nutrients and SOC (Table 4) concurred with Cao *et al*.^57^. Since SOC is usually closely coupled with N and P^58^, potential increases in AN and AP may depend on soil organic matter decomposition^59^. TP and AP were also positively related to SOC as well as to TN and AN. Although the exact cause of these relationships is not yet clear, a plausible explanation is that P can be fixed relatively slowly by clay minerals, carbonates and soil organic matter as part of biochemical cycling, and a higher P could further fix N and support greater accumulation of organic matter^57^. TK and AK were not related to SOC, which was consistent with Liu *et al*.^60^. The reasons for this lack of a relationship require further study.

Based on the PCA (Fig. 2), TN and *θ* were the dominant factors, which was consistent with Tian *et al*.^61^, since they have important roles in increasing SOC^62^. As biotic and abiotic interactions strongly impact ecological processes, many studies have explored the effects of these interactions on variables (e.g.^63,64^). For example, Wu *et al*.^65^ found that grassland community coverage and above- and below-ground biomass were related to the interaction of plant diversity and *θ*, and Merino *et al*.^66^ found that soil carbon level was correlated with the interactions of plants, microorganisms, and mineralogy. However, no significant interaction was shown between *θ* and other variables in the present study. Further research is required to support the results of the current investigation.

As described above, TCS was highest at the lowest elevation. This may be due to good soil conditions, specifically good physical properties (Tables 1 and 2). However, as elevation increased, TCS did not present a clear trend, which countered the findings of Seedre *et al*.^21^. In their study, they found that a significant decrease (*P* < 0.05) in TCS occurred with increasing elevation. This suggests that elevation has a complicated effect on TCS and has no common trend. As TC_BMag_, TC_BMbg_, and C_litter_ were similar among three stand ages (Table 2), it can be concluded that TCS was determined by SCS, as SCS was the largest C pool (69.2% of TCS) in the whole forest ecosystem.

### Implications for forest management

Generally, dragon spruce plantations in the upper reaches of the Bailongjiang River basin were water conservation forests. Interestingly, the capacity for water conservation and TCS were lower in the Y3 age group than the younger age groups. The fact that trees of a certain maturity level have reduced biomass production and merely maintain their own survival indicates that old forests could become a net source of carbon or be carbon neutral^20,21^. Since forest landscape management has the potential to influence the net carbon sink, governments must address the current situation and take measures to minimize carbon losses and maintain water resources when considering the economic benefits of forests^22^. In the twenty-first century, any landscape management strategy should be integrated with economic and environmental dimensions to create a sustainable long-term plan^8,67^. For example, using a landscape management approach in the Missouri Ozarks, even-aged and uneven-aged forests are being planted to conserve biodiversity, improve wildlife habitat, enhance forest health and sustain timber production^26^. In the central hills of Nepal, the rotation age for forest plantations was determined to be between 40 and 45 years old^68^, as older forests lost the potential for enhancing CS^23^. The present research suggests that dragon spruce plantation forests of the Y_3_ age group should be thinned and seedlings planted in order to renew the forest. These measures would not only support the realization of the economic value of older forests, but also increase TCS and maintain water resources in the study area.

## Conclusions

The dragon spruce plantation forest located along the upper reaches of the Bailongjiang River was found to be carbon negative as stand age increased. The average TCS of the three age groups was 255.65 Mg C ha^−1^. While no difference was observed among other carbon pools of the three age groups, the Y_3_ age group with a TCS of 174.60 Mg C ha^−1^ showed greater carbon losses than the Y_1_ age group (323.64 Mg C ha^−1^), due to significant differences in SCS and TCS. This suggests that even-aged (about 30 years) forest spruce plantation forests should be preferred for forest landscape management in this region.

The dragon spruce stands in this mountainous region were already mature and since carbon loss continues when growth stagnates, keeping tree plantations young through rotations may be a useful measure to increase TCS in the long term. Policy-makers would have to create legislation to protect the region at the landscape level in order to improve the efficiency of forest management and the region’s adaptability to future climate change.

## Materials and Methods

### Study area

**Figure 4:**
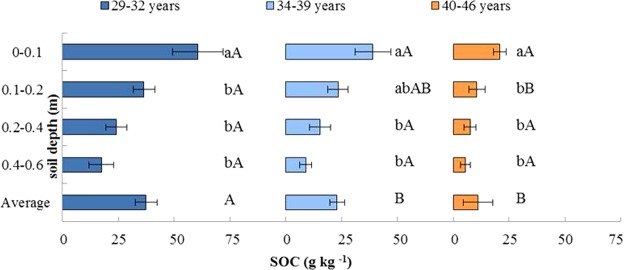
Study area in the Bailongjiang River Basin, China.

Located in the northeastern part of the Qinghai-Tibetan Plateau, west of the Qinling Mountains, dragon spruce plantations in the upper reaches of the Bailongjiang River (latitude 33°04′N-35°09′N, longitude 102°46′E-104°52′E, Fig. 4) are among key protected areas that provide water and reduce soil erosion for the Yangtze River basin. The Bailongjiang River basin covers roughly 3.3 × 10^7^ ha and has an annual mean runoff of about 4.0 × 10^9^ m^3^ y^−1^. The upper reaches of the Bailongjiang River are situated in a zone that crisscrosses northern subtropical and warm temperate climatic zones, along with semi-humid mountain ravines that receive heavy and largely concentrated rainfall. Over the past 50 years, the air temperature in this region has increased significantly. There has also been a slight reduction in precipitation and a significant reduction in surface run-off^69^.

### Field sampling

In 2013, based on the local Forestry Bureau’s records of planting times, the dragon spruce plantations in this region were determined to be between 29 to 46 years old, and considered to be mature^70^. As a longer chronosequence-based scale would more accurately reflect the variation of TCS with stand age^9,21^, tree age was divided into three categories (i.e. 29–32 years, Y_1_; 34–39 years, Y_2_; 40–46 years, Y_3_). According to local officials, dragon spruce trees were often planted with other species, such as *Betula albosinensis*, and *Larix gmelinii*, meaning that areas of pure dragon spruce forest were relatively small. Furthermore, a majority of the pure stands were located at three elevations: about 2520 m, 2640 m and 2848 m. To ensure comparability among the sample sets in terms of the other environmental covariates, plots of each age group were established at each elevation. However, there was an uneven distribution of plots at each elevation due to an uneven distribution of pure stands with different ages. This protocol was used in previous studies^71,72^, as it precludes topographical factor effects (e.g., the effects of altitude and slope).

Stand density (SD), diameter at breast height (DBH), and canopy density (CD, the ratio of the sum of the crown area of all trees within a plot to the plot’s area^72^), were measured in each plot (20 m × 20 m). To estimate the fresh weight of the trees, one standard dragon spruce was cut down in each plot and the Monsic Layered cut method was used to section and weigh biomass^35^. Because this region is a natural conservation area, further cutting of trees was not permitted. In addition, the difference in the DBH of trees within any plot in each age group was small (Table 1). The total above-ground portions of the tree were weighed by dividing them into sections of particular lengths. For understory vegetation with shrubs, three 2 m × 2 m quadrats were sampled along the diagonal of the plot, at ends and midpoint. For herbaceous layers, 1 m × 1 m quadrats were similarly used. For the litter layer, a 0.1 m × 0.1 m quadrat was used at the midpoint of the diagonal in all plots.

In each quadrat, vegetation biomass composed of leaves and branches of shrubs and herbs and litter were collected. The soil profile was then excavated to a depth of 0.6 m, with three replications sampled along the diagonal of the quadrat (at the ends and midpoint). Soil samples were taken at depths of 0–0.1 m, 0.1–0.2 m, 0.2–0.4 m, and 0.4–0.6 m, using a cutting ring (volume, 1.0 × 10^−4^ m^3^), and divided into two parts. Compared with other methods, this approach provided researchers with a better comparison of soil properties at multiple depths^39,63,71,73^. One part of each soil sample was used to measure *ρ*, *f* and *θ*, while the other part was used to measure pH, SOC, TN, TP, AN, AP and AK in each layer of the soil profile.

### Sample analysis

The fresh herb and litter biomass were oven-dried at 80 °C to a constant weight over a 24-hour period. Dragon spruce trunks were cut into 1 m segments and the crown was divided into leaves and branches with branches further categorized into thin (<0.01 m) and coarse (>0.01 m). All were weighed. Following this, the total dragon spruce biomass (kg ha^−1^) was estimated using the following equation^74^:

1 $${{\rm{BM}}}_{\mathrm{all}\mathrm{Pa}}={{\rm{BM}}}_{\mathrm{one}\mathrm{Pa}}\times {\rm{SD}}$$

where, BM_all Pa_ is the total above-ground biomass of dragon spruce in a given plot, BM_one Pa_ is the above-ground biomass of one average dragon spruce in a given plot, and SD is the stand density.

To convert the fresh weight of tree and shrub biomass to dry biomass (kg ha^−1^), a default moisture content of 30–40% can be used^75^. In the present study, the median value of moisture content, 35%, was used.

The below-ground tree biomass estimation was based on a “root-to-shoot” ratio of 0.2^76^. The largest “root-to-shoot” shrub ratio was 0.93 and the smallest was 0.25^77^. In this study, we used the median shrub “root-to-shoot” ratio of 0.59 to estimate below-ground shrub biomass. The below-ground herbaceous biomass was established as an average of 82% of the total herbaceous biomass^78^, and the ratio was found to be 4.6.

Soil pH was measured with a standard pH meter using a 2.5:1 water: air-dried soil ratio. The SOC (g kg^−1^) was determined by wet dichromate oxidation of a homogenized air-dried soil subsample (0.2 g), followed by titration with FeSO_4_^73^. Both TN (g kg^−1^) and TP (g kg^−1^) were measured using a Smartchem 140 (AMS/Westco, Italy) chemical analyzer^71^. AN (mg kg^−1^) was measured by the Kjeldahl method^62^, while TK (g kg^−1^), AP (mg kg^−1^) and AK (mg kg^−1^) were determined using the method adopted by Verma *et al*.^79^.

SCS (Mg ha^−1^), *ρ* (Mg m^−3^), *θ* (%), and *f* (%) were calculated using the following equations^62^:

2 $${\rm{SCS}}=[{\rm{SOC}}]\cdot \rho \cdot {\rm{T}}$$

3 $$\rho =\frac{{{\rm{m}}}_{{\rm{d}}}}{{{\rm{V}}}_{{\rm{s}}}}$$

4 $$\theta =\frac{{{\rm{m}}}_{{\rm{f}}}-{{\rm{m}}}_{{\rm{d}}}}{{{\rm{m}}}_{{\rm{d}}}}\cdot 100 \% $$

5 $$f=(1-\frac{\rho }{{{\rm{G}}}_{{\rm{s}}}})\cdot 100 \% $$

where, [SOC] is the concentration of C in the soil (%), T is the soil layer thickness (m), V_s_ is the volume of soil (1 × 10^−4^ m^3^), m_d_ is the dry weight (mass) of soil (g), m_f_ is the fresh weight (mass) of soil (g), and G_s_ is the soil particle density (Mg m^−3^).

Accordingly, TCS was calculated as:

6 $${\rm{TCS}}={{\rm{TC}}}_{{{\rm{BM}}}_{{\rm{ag}}}}+{{\rm{TC}}}_{{{\rm{BM}}}_{{\rm{bg}}}}+{{\rm{C}}}_{{\rm{litter}}}+{\rm{SCS}}$$

where, TC_BMag_ is total CS in above-ground dry biomass, TC_BMbg_ is total CS in below-ground dry biomass, and C_litter_ is CS in litter dry biomass.

The above- and below-ground dry biomass and litter dry biomass were converted into carbon by multiplying by a factor of 0.47, as adopted by the MFSC^80^.

### Data analysis

Data were analyzed using SPSS 22.0 (SPSS Inc. Chicago, USA) statistical software and expressed as the mean value ± standard deviation. One-way analysis of variance (ANOVA) was applied to determine the differences in the measured variables for the three stand ages. A two-tailed least significant difference test was conducted when significant differences were detected by the ANOVA process. The Spearman correlation was used to identify the possible relationships between soil physicochemical properties and the vegetation characteristics. To eliminate any possible redundancy among the biological factors and the relevant soil property variables, this study also adopted principal components analysis (PCA), which aimed to reduce data dimensionality while capturing most of the variations in the dataset^61^. The Origin Pro 9.0 software was used to draw graphs and a probability threshold of P < 0.05 was applied as the critical threshold for the significance level to determine differences in the studied variables.

## Data Availability

The datasets generated and/or analyzed during the current study are available from the corresponding author on request.
